# Comprehensive Transcriptomic Analysis of Auxin Responses in Submerged Rice Coleoptile Growth

**DOI:** 10.3390/ijms21041292

**Published:** 2020-02-14

**Authors:** Yu-Sian Wu, Chin-Ying Yang

**Affiliations:** 1 Department of Agronomy, National Chung Hsing University, Taichung 40227, Taiwan; celebrate199094@gmail.com; 2 Pervasive AI Research (PAIR) Labs, Hsinchu 30010, Taiwan

**Keywords:** rice coleoptile, transcriptomic analysis, submergence, auxin signaling, hormone

## Abstract

Cultivating rice in wet or water direct seeding systems is simple and time and labor efficient. Rice (*Oryza sativa*) seeds are a unique cereal that can germinate not only when submerged, but also in anoxic conditions. Many complicated hormone signals interact in submerged seed germination. Ethylene is involved in rice coleoptile elongation, but little is known regarding the role of auxin signaling under submergence. This study demonstrated that the coleoptile is shorter and curlier when submerged with 2,3,5-triiodobenzoic acid (TIBA). In transcriptomic analysis, 3448 of the 31,860 genes were upregulated, and 4360 genes were downregulated with submergence and TIBA treatment. The Gene Ontology function classification results demonstrated that upregulated differentially expressed genes (DEGs) were mainly involved in redox, stress, and signal transduction, whereas the down-regulated DEGs were mainly involved in RNA transcription, stress, and development. Furthermore, auxin signaling involved in the carbohydrate metabolism pathway was demonstrated while using transcriptomic analysis and confirmed in a quantitative real-time polymerase chain reaction. In addition, the transcript levels of development-related genes and mitochondria-electron- transport-related genes were regulated by auxin signaling under submergence. Auxin signaling was not only involved in regulating rice coleoptile elongation and development, but also regulated secondary metabolism, carbohydrate metabolism, and mitochondria electron transport under submergence. Our results presented that auxin signaling plays an important role during rice coleoptile elongation upon the submergence condition and improving the advance of research of direct rice seeding system.

## 1. Introduction

Rice is among the most valuable cereal crops in the world. The rice coleoptile’s ability to grow in underwater environments has practical value for rice cultivation. Direct seeding of rice is performed by airplane over a field with a water depth of 35 cm. The seeds germinate under water, and the coleoptiles grow up to the water surface within a few days [1]. The direct seeding of rice is becoming popular and more crucial for rapid and uniform germination. Seed germination is a complex physiological process that must be tightly regulated to maximize plant survival by using various environmental cues [2,3]. Global climate change causing severe rain can negatively affect the seed germination, seedling growth, and production yields of rice [4]. Clarifying and establishing the regulation mechanism of submerged rice coleoptile growth is crucial for improving seed germination in paddy fields.

The involvement of phytohormones in regulating rice coleoptile elongation under different environmental conditions is complicated, and hormones may contribute to forming an effector system for environmental factors for the control of coleoptile elongation in rice [5,6,7]. Ethylene promotes rice coleoptile elongation under submerged conditions. In addition, auxin is involved in coleoptile elongation [8] and it promotes cell division and meristem maintenance. Auxin treatment induced rapid cell elongation in coleoptile and hypocotyl segments within minutes [9]. Auxin affects growth and development in plants by altering gene expression. Many auxin-responsive genes have been characterized in plants. Studies have indicated that coleoptile phototropism1 (CP1) involvement in the phototropism of coleoptiles is achieved by lateral auxin translocation and subsequent growth redistribution [10]. Auxin response factor1 (OsARF1) was positively correlated with auxin-dependent differential growth in rice coleoptiles [11]. Ion transport activity was also involved in promoting the auxin-induced growth of maize coleoptile segments [12].

Although the phytohormone auxin is involved in regulating rice coleoptile elongation, little is known regarding the roles of auxin in coleoptile growth responses in submerged conditions. This study compared the transcriptomic profiles of submerged rice coleoptiles and those that were treated with 2,3,5-triiodobenzoic acid (TIBA), which acts as an auxin polar transport inhibitor. According to our data, auxin signaling was involved in the cell division and tropism of submerged rice coleoptiles. In addition, auxin signaling played a central role in carbohydrate consumption during rice coleoptile elongation in the submerged condition. Therefore, our data provide important insight into the development of coleoptiles at the post-germination stages in the wet direct-seed system under submerged conditions.

## 2. Results

### 2.1. Submerged Rice Coleoptile Elongation Affected by Impeded Auxin Signaling

**Figure 1:**
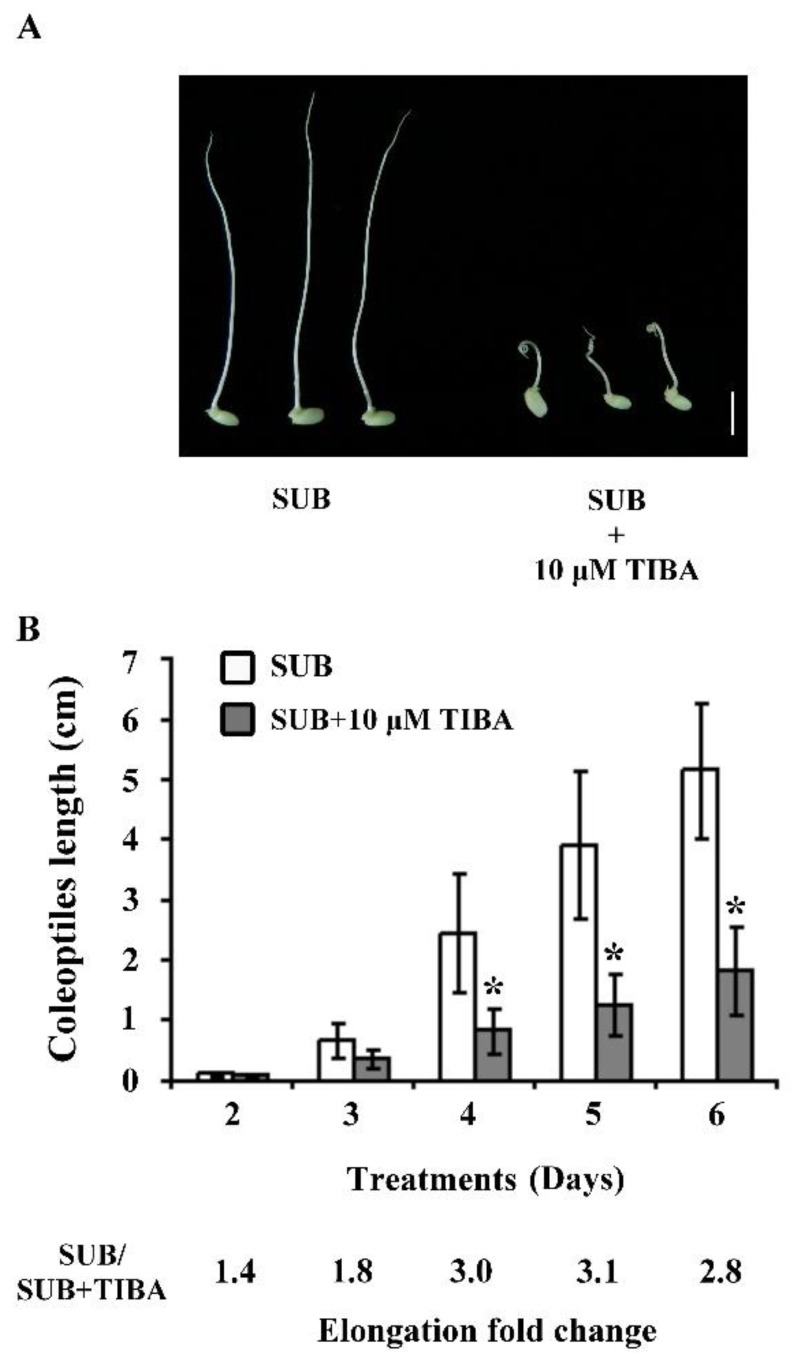
The effects of auxin polar transport inhibitor 2,3,5-triiodobenzoic acid (TIBA) on submerged rice coleoptiles. (**A**) Rice seeds after submergence (SUB) and submergence combined with 10 µM TIBA (SUB + TIBA) for five days. (**B**) Lengths of rice coleoptiles after SUB and SUB + TIBA treatment for 2–6 days. Bar = 1 cm. Data expressed as average values ± standard deviations from six biologically independent experiments. * *p* < 0.05 versus value in submergence treatment (Student’s *t*-test).

The polar auxin transport inhibitor TIBA was added to the combined submergence treatment to investigate the effect of auxin on rice coleoptile elongation under submerged conditions. The elongated rice coleoptile is appearing after submergence for five days, and the coleoptiles display significantly shorter and curlier after SUB + TIBA for five days (Figure 1A). The length of coleoptiles from seeds germinated and grown under the submergence only condition for two, three, four, five, and six days were 0.11, 0.66, 2.45, 3.92, and 5.16 cm, respectively, whereas the average lengths of rice coleoptiles under SUB + TIBA were 0.08, 0.36, 0.82, 1.26, and 1.82 cm, respectively (Figure 1B). The results indicated that auxin signaling is involved in rice coleoptile elongation and tropism under submerged conditions.

### 2.2. RNA Sequencing and Functional Annotation of DEGs in Rice Coleoptiles 

**Figure 2:**
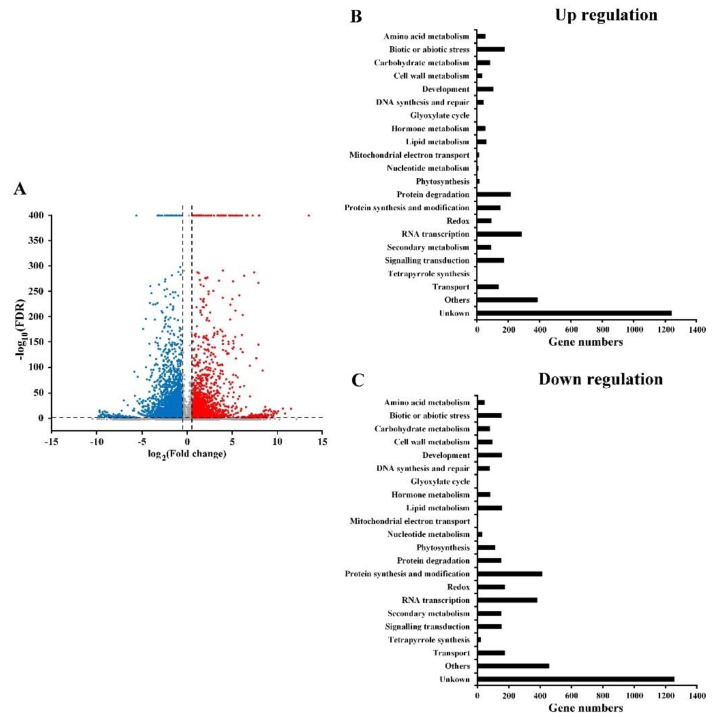
Volcano plots and Gene Ontology (GO) classification of differentially expressed genes in 10 μM TIBA treatment compared with submergence treatment. (**A**) Volcano plots of differentially expressed genes between between SUB and SUB + TIBA treatment. The log2 fold change (FC) of each gene on x-axis and log10 false discovery rate (FDR) of each gene on y-axis. The log2 fold change ≥ 0.584 (up-regulated genes) and ≤−0.584 (down-regulated genes) and FDR less than 0.01 were considered as significantly differentially expressed gene. (**B**) GO classification of differentially expressed genes in upregulation. The filter condition was fold change ≥ 1.5 and FDR less than 0.01. (**C**) GO classification of the differentially expressed genes in downregulation. The filter condition was fold change ≤ 0.66 and FDR less than 0.01. Table S3 presents the complete list of differentially expressed genes in GO classification.

We performed RNA sequencing (RNA-seq) analysis to clarify the roles of auxin in elongating submerged coleoptiles to investigate auxin signaling regulation in rice coleoptile growth under submergence. Rice coleoptile growth under the SUB and SUB + TIBA conditions after five days was collected for RNA-seq. In total, 11,338,976 and 11,213,701 clean reads were generated for SUB and SUB + TIBA and mapped to 80.92% and 79.7% unique genes in the rice genome, respectively (Table S1). The volcano plot in Figure 2 presents the comparison results of the UniGene expression levels in individual mRNA samples that were relatively enriched or depleted according to the region per million mappable reads (RPKM) value. In the SUB + TIBA condition, the expression levels of 3448 of the 31,860 genes were upregulated, whereas the remaining 4360 genes exhibited lower expression (Figure 2A). The GO classifications of DEGs in up- and down-regulation in the SUB + TIBA treatment were mapped against the KEGG database and then classified into 22 subcategories of biological processes. The upregulated DEGs were mainly involved in RNA transcription (8.3%; GO:0006351), protein degradation (6.2%; GO: 0019538), and biotic or abiotic stress (5.2%; GO: 0006950; Figure 2B), whereas the downregulated DEGs were mainly involved in protein synthesis and modification (9.5%; GO: 0044267), RNA transcription (8.8%; GO:0006351), and transport (4.1%; GO: 0006810; Figure 2C). 

We identified 23 upregulated genes and 39 downregulated genes, which were filtered by a fold change (FC) of ≥ 200 or ≤ 0.005, in coleoptiles that were submerged with TIBA when compared with those submerged without TIBA. In the upregulated genes, we identified a heat shock protein (*Os06g0195800*) that was involved in protein refolding, redox-related genes, including 1-cysperoxiredoxin (*Os07g0638300*), cytochrome P450 (*Os01g0628000*), and clutathione S-transferase (*Os03g0785900*, *Os09g0367700*, *Os10g0527400*), and responsive to abscisic acid (ABA) 16D protein (*Os11g0453900*), which is involved in ABA response. We also identified E3 ubiquitin-protein ligase (*Os09g0243200*, *Os08g0126000*) that was involved in the protein ubiquitination pathway and a small GTPase (*Os06g0225000*) involved in G-protein signaling (Table 1). Among the downregulated genes, we identified transcription factors in the AP2 domain containing ethylene response transcription factors (*Os01g0968800*, *Os02g0781300*, and *Os09g0522200*), myb-like DNA-binding domain containing protein (*Os05g0442400*), WRKY transcription factor (*Os01g0821300*), and the lateral organ boundaries domain (LBD) family transcription factor (*Os12g0106200*). Calmodulin (*Os11g0105000*, *Os12g0104900*) is a crucial Ca^2+^ sensor in signal transduction in plants. In addition, we identified a highly sensitive auxin-responsive protein (*Os09g0437100*) and a bidirectional sugar transporter (*Os01g0606000*) that were associated with development response. The plant transporter included a proton-dependent oligopeptide transporter (*Os05g0338933*) and aquaporin (*Os02g0658100*), which were significantly downregulated in our analysis (Table 2).

### 2.3. The DEGs in Metabolism Process Regulated by Auxin Signaling in Submerged Rice Coleoptiles 

**Figure 3:**
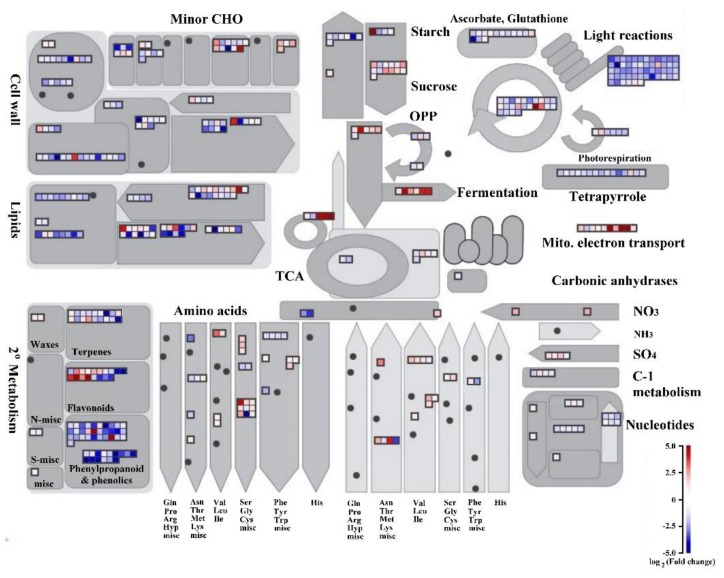
Metabolism overview of differentially expressed genes between submergence treatment and submergence treatment with polar auxin transport inhibitor in coleoptiles, illustrated using MapMan. The log2 fold change (FC) color scale ranges from −5 to 5. Red and blue squares represent up- and down-regulated responses in coleoptiles under SUB + TIBA when compared with SUB only. Minor carbohydrate (Minor CHO), Miscellaneous enzyme (Misc), Oxidative pentose phosphate (OPP), Citric acid (TCA), One carbon metabolism (C-1 metabolism). Table S4 presents the complete list of genes and calculated FC.

We used MapMan to visualize the metabolic pathways to assess the effect of auxin signaling in submerged rice coleoptiles. We identified 504 DEGs involved in metabolic pathways, of which 159 were upregulated and 345 were downregulated. Both comparison treatments revealed that 63, 69, 87, 53, 53, 6, 6, 5, 8, and 10 genes were involved cell-wall metabolism, lipid metabolism, secondary metabolism, amino acid metabolism, carbohydrate metabolism, glycolysis, fermentation, oxidative phosphorylation, tricarboxylic acid (TCA) cycle, and mitochondrial electron transport, respectively. We identified strong regulation in several cell-wall-modification–related genes, including genes encoding an expansin (*Os07g0496250*) and xyloglucan endotransglycosylase (*Os03g0108300* and *OsXTH19*; *Os10g0545500*; and, *OsXTH20*). Cell-wall-degradation–related genes, including those encoding beta-d-xylosidase (*Os11g0291000* and *Os11g0297300*), which is the enzyme that is responsible for the cleavage of the xylem backbone, were downregulated in SUB + TIBA treatment. For lipid degradation, lipase (*Os11g0299300* and *Os05g0153300*) was downregulated. Among the phospholipase D family proteins, which have roles in hormone signaling and environmental stress responses, *Os02g0120200* was upregulated, but *Os06g0604300* was downregulated in SUB + TIBA treatment. In the secondary metabolism pathway, most of the genes encoding laccase (*Os03g0273200*, *Os05g0458600*, *Os05g0458300*, *Os01g0374600*, *Os03g0297900*, *Os01g0850800*, and *Os11g0108700*), which play key roles in plant growth and defense responses, were downregulated in SUB + TIBA treatment (Figure 3 and Table S4). These observations demonstrated that auxin signaling is involved in the metabolic processes of submerged rice coleoptiles.

### 2.4. Transcript Levels of Development-Responsive Genes Regulated by Auxin Signaling in Submerged Rice Coleoptiles

The coleoptiles submerged with TIBA were shorter and curlier than those that were submerged without TIBA (Figure 1). Therefore, we analyzed the transcript levels of development-responsive genes in submerged coleoptiles. Two genes were associated with cell division: *Os01g0281200* encoding G2/mitotic-specific cyclin-B1-3 (OsCYCB1-3) and *Os06g0236600* encoding G1/S-specific cyclin-D1-1 (OsCYCD1-1). Both of the cell-division-related genes were downregulated when submerged with TIBA. In addition, three genes were associated with vascular auxin transport (walls are thin 1, WAT1), which is involved in regulating intracellular auxin homeostasis in plants. The transcript levels of *Os01g0803300*, *Os04g0422300*, and *Os06g021000* were significantly downregulated in SUB + TIBA treatment. Furthermore, most sugar transport genes, such as *Os01g0606000* (*OsSWEET6a*), *Os12g0476200* (*OsSWEET13*), *Os11g0508600* (*OsSWEET14*), and *Os01g0700100* (*OsSWEET2b*), were downregulated in SUB + TIBA treatment (Table 3).

**Figure 4:**
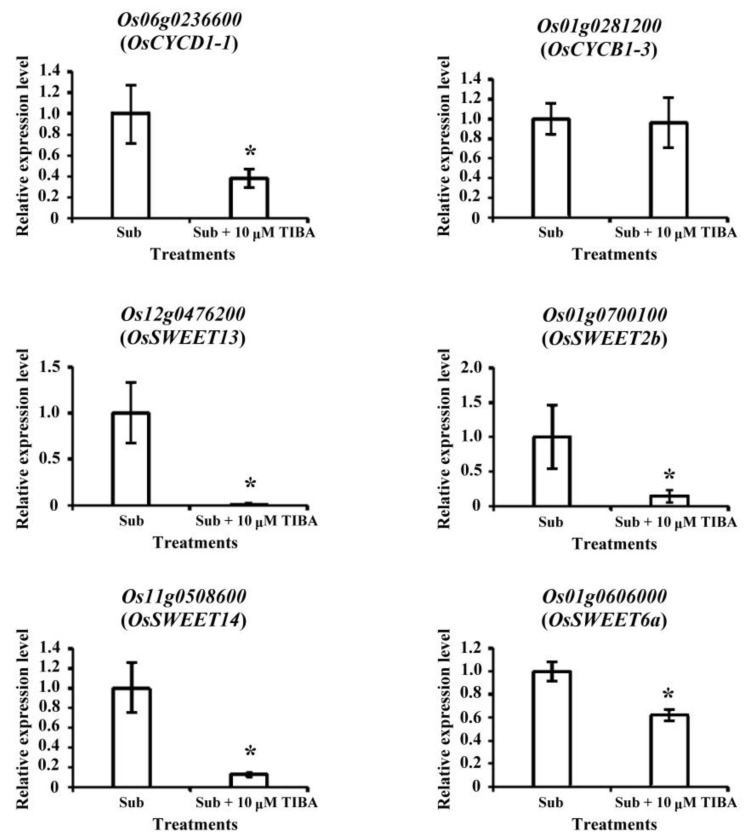
Transcript levels of development-associated genes regulated by auxin signaling in submerged coleoptiles. Total RNA was isolated from the coleoptiles after submergence treatment with or without 10 µM 2,3,5-triiodobenzoic acid (TIBA) for 5 days. Relative expression levels of *Os06g0236600* (*OsCYCD1-1*), *Os01g0281200* (*OsCYCB1-3*), *Os12g0476200* (*OsSWEET13*), *Os01g0700100* (*OsSWEET2a*), *Os11g0508600* (*OsSWEET14*), and *Os01g0606000* (*OsSWEET6b*) were determined while using quantitative RT-PCR. These genes were calculated and normalized with ubiquitin gene (*Os02g0634800*) as an internal standard. Values are presented as mean ± standard deviation based on 30 coleoptiles of each treatment obtained from six biologically independent experiments. * *p* < 0.05 versus the submergence treatment value (Student’s *t*-test).

We confirmed the transcript levels of the development-responsive genes by using qRT-PCR. The transcript levels of *OsCYCD1-1*, *OsSWEET13*, *OsSWEET2b*, *OsSWEET14*, and *OsSWEET6a* were reduced. Only *OsCYCD1-3* had no significant difference in SUB + TIBA treatment (Figure 4).

### 2.5. Transcript Levels of Carbohydrate-Metabolism-Associated Genes Regulated by Auxin Signaling in Submerged Rice Coleoptiles

**Figure 5:**
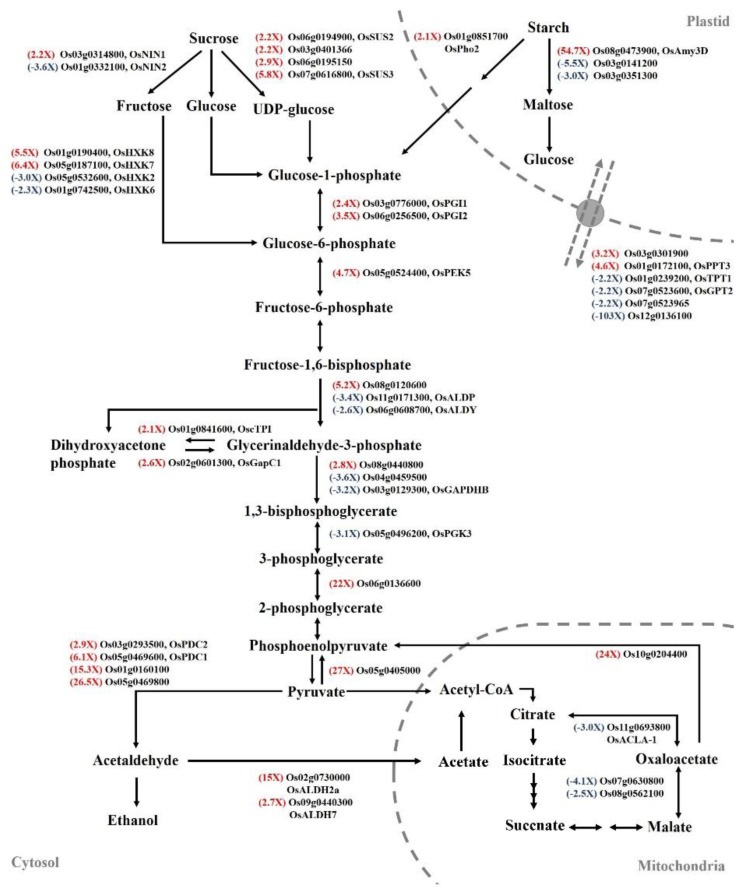
Differentially expressed genes involved in starch degradation, glycolysis, and fermentation pathways. Red text represents fold change (FC) and significant upregulation in SUB + TIBA treatment compared with SUB treatment. Blue text represents FC and significant downregulation. The qRT-PCR profile confirmed that the transcript levels of carbohydrate-metabolism–related genetic expression were similar to the RNA-Seq results. The transcript levels of amylase (*Os08g0473900*), sucrose synthase (*Os07g0616800*), hexokinase (*Os05g0187100* and *Os01g0190400*), fructose-bisphosphate aldolase (*Os08g0120600*), phosphoenolpyruvate carboxykinase (*Os10g0204400*), lactic acid dehydrogenase (*Os02g0730000*), pyruvate decarboxylase (*Os05g0469600*), and phosphoenolpyruvate/phosphate translocator (*Os01g0172100*) were induced, and plastidic phosphate translocator (*Os12g0136100*), fructose-bisphosphate aldolase (*Os11g0171300*), and glyceraldehyde-3-phosphate dehydrogenase (*Os03g0129300*) were reduced under SUB + TIBA treatment (Figure 6). Our results indicate that auxin signaling is involved in energy production processes under submerged conditions. The red word showed upregulated genes, whereas the blue word showed downregulated genes.

In transcriptomic analysis, genes that were related to carbohydrate metabolism exhibited significant expression changes when SUB + TIBA condition (Figure 5). Most of the genes were presented up-regulation in carbohydrate metabolism pathways, including amylase (*Os08g0473900*; *OsAmy3D*), which catalyzes the starch into maltose, and sucrose synthase (*Os06g0194900*, *Os03g0401366*, *Os06g0195150*, and *Os07g0616800*), which has a role in producing UDP-glucose. In the glycolysis pathway, glucose-6-phosphate isomerase (*Os03g0776000*, *Os06g0256500*), 2-phosphoglycerate dehydratase (*Os06g0136600*), and pyruvate orthophosphate dikinase (*Os05g0405000*) also exhibited an upregulation in the SUB + TIBA condition. Under oxygen-limited conditions, fermentation produces nicotinamide adenine dinucleotide (NAD^+^) for maintaining glycolysis and converts the glycolysis-derived pyruvate into lactate, ethanol, or acetate. We identified pyruvate decarboxylase (*Os03g0293500*, *Os05g0469600*, *Os01g0160100*, and *Os05g0469800*), which catalyzes pyruvate into acetaldehyde, and lactic acid dehydrogenase (*Os02g0730000* and *Os09g0440300*), which catalyzes pyruvate into lactate. The TCA cycle–related genes, ATP-citrate synthase (*Os11g0693800*), and nodule-enhanced malate dehydrogenase (*Os07g0630800* and *Os08g0562100*) were downregulated, but phosphoenolpyruvate carboxykinase (*Os10g0204400*) was upregulated when receiving TIBA treatment (Figure 5).

### 2.6. Transcript Levels of Mitochondrial-Electron-Transport–Associated Genes Regulated by Auxin Signaling in Submerged Rice Coleoptiles

**Figure 7:**
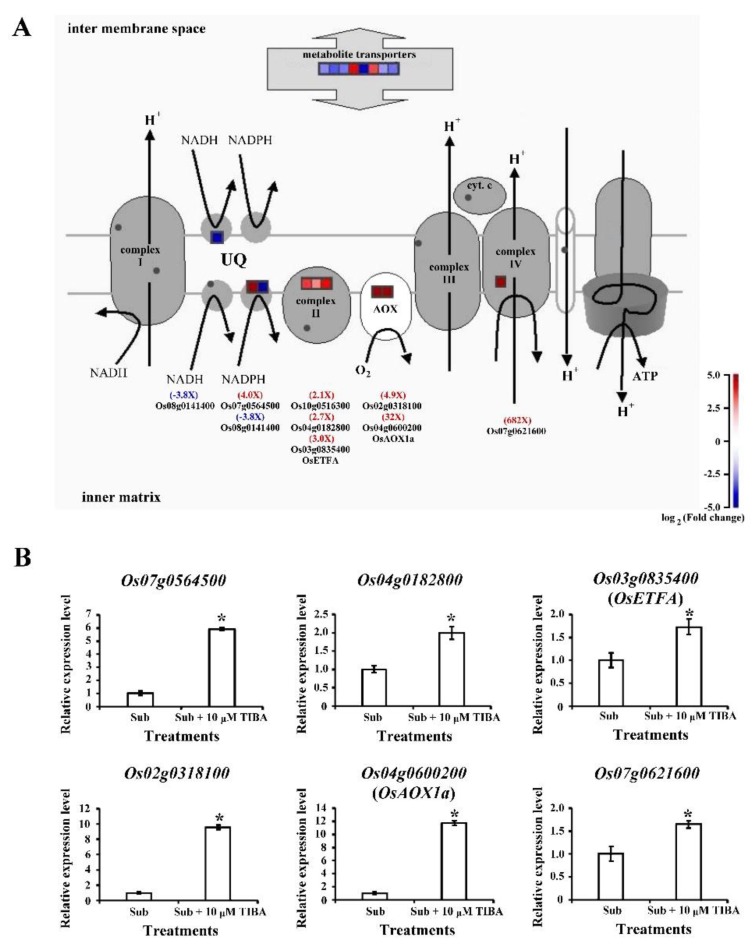
Transcript levels of mitochondrial-electron-transport–associated genes regulated by auxin signaling in submerged coleoptiles. (**A**) The differentially expressed genes of mitochondrial electron transport in the SUB and SUB + TIBA treatments in coleoptiles were illustrated using MapMan. The log2 fold change color scale ranges from −5 to 5. Red and blue squares represent up- or down-regulated response, respectively, in coleoptiles submerged with 10 μM TIBA compared with those submerged without TIBA treatment. (**B**) Relative expression levels of mitochondrial-electron-transport–associated genes were determined using a quantitative real-time polymerase chain reaction. Total RNA was isolated from the coleoptiles after 5 days of submergence treatment with or without 10 µM TIBA. The expression levels of *Os04g0182800*, *Os02g0318100*, *Os07g0564500*, *Os03g0835400* (*OsETFA*), *Os04g0600200* (*OsAOX1a*), and *Os07g0621600* were determined. These genes were calculated and normalized with ubiquitin gene (*Os02g0634800*) as an internal standard. The red word showed upregulated genes, whereas the blue word showed downregulated genes. The values are presented as mean ± standard deviation based on 30 coleoptiles for each treatment obtained from six biologically independent experiments. * *p* < 0.05 versus the submergence treatment value (Student’s *t*-test).

Mitochondrial electron transport flux was reduced in low-oxygen conditions [13]. We identified the DEGs involved in mitochondrial electron transport and then performed a qRT-PCR analysis to investigate whether auxin signaling is involved in cellular respiration processes. Genes encoding succinate dehydrogenase (mitochondrial complex II, *Os04g0182800*, and *Os03g0835400*), which is involved in reactive oxygen species (ROS) production, were upregulated. The genes encoding alternative oxidase, such as *Os02g0318100* and *Os04g0600200*, were upregulated under SUB + TIBA treatment, where the function was transferred electrons from ubiquinone to oxygen, thereby preventing ROS production from the ubiquinone pool in mitochondria (Figure 7A,B).

## 3. Discussion

Rice germination under submergence was characterized by coleoptile elongation and delayed radicle emergence [14]. Under oxygen deprivation, the primary leaf fails to elongate, and a much longer coleoptile is produced. The elongated coleoptiles can reach the water surface and, subsequently, provide a path for O_2_ diffusion to the submerged parts [15,16]. The auxin phytohormone regulates many critical growth and developmental processes in rice. Auxin is synthesized in plant cells and actively transported between cells through polar transport. Auxin mediates rice root system growth, tiller number change, leaf shape growth, and grain size [17]. Cell division and the cell cycle are regulated by coleoptile elongation under submerged conditions. The base and tip parts of coleoptiles exhibited different transcriptomic profiles under hypoxia [18]. In our study, rice coleoptile elongation was significantly reduced for 3.0 fold change under submergence treatment with an auxin polar transport inhibitor compared with submergence only in four days treatment (Figure 1). The coleoptiles had a shorter and curlier phenotype in the submergence with auxin polar transport inhibitor condition, indicating that auxin signaling is involved in the rice coleoptile elongation response.

ROS participate in plant stress responses and the ROS scavenging enzyme that are involved in the detoxification of excess ROS to maintain cellular redox homeostasis. Glutathione S-transferases are ubiquitous enzymes that play a key role in cellular detoxification. The transcript levels of *Os03g0785900* and *Os09g0367700* were enhanced by herbicide treatment in *Avenafatua* plants [19]. Under drought stress, the transcript level of *Os10g0527400* increased in mutant *osrbohA* as compared with wild type [20]. Our results revealed significantly upregulated genes, such as 1-cysperoxiredoxin (*Os07g0638300*), cytochrome P450 (*Os01g0628000*), and glutathione S-transferase (*Os03g0785900*, *Os09g0367700*, and *Os10g0527400*), involved in redox processes (Table 1). It is demonstrated that those ROS scavenging enzymes that were involved in rice coleoptile elongation were regulated by auxin signaling during submergence stress. Our data shown significantly downregulated genes in coleoptiles under SUB + TIBA treatment including ethylene response transcription factor (*Os01g0968800*, *Os02g0781300*, and *Os09g0522200*), myb-like DNA-binding domain containing protein (*Os05g0442400*), WRKY transcription factor (*Os01g0821300*), and LBD family transcription factor (*Os12g0106200*) (Table 2). The study indicated that rice seedlings with *Os01g0968800* line overexpression had an increased tolerance to salt stress [21]. The transcript levels of *Os01g0968800* and *Os09g0522200* were significantly increased in rice seedlings under low-temperature stress [22]. *Os12g0106200* encoded for LBD proteins that are essential for adventitious root formation and it was upregulated in rice seedling roots under water-deficient conditions [23]. Our data presented that these genes were not only involved in abiotic stress, but also regulated by auxin signaling under submergence. In addition, the genes encoding laccase (*Os03g0273200*, *Os05g0458600*, *Os05g0458300*, *Os01g0374600*, *Os03g0297900*, *Os01g0850800*, and *Os11g0108700*) were downregulated under SUB + TIBA treatment (Figure 3 and Table S4). Reduced expression levels of laccases result in low lignification phenotypes, increased stem thickness, and they have a tendency to lodge and reduce stem and leaf length [24]. Our results further indicated that laccase genes are regulated by auxin signaling and affected the coleoptile appearance change under submergence.

The auxin phytohormone is involved in many stages of plant development, including root growth, apical dominance, and seed germination [25]. Auxin-induced seed dormancy and delayed seed germination are affected by ABA and GA signal transduction in wheat and *Arabidopsis* plants [26,27]. We obtained shorter and curlier coleoptile phenotypes under TIBA treatment as compared with submergence treatment only (Figure 1A). The transcript levels of the cell-cycle-related gene (*Os06g0236600*) were significantly downregulated. The cell-cycle-related gene *Os06g0236600* encodes cyclin-D1-1, which is a rate-limiting component in the G1 phase. In rice, members of the CDK proteins regulate root and shoot meristem cell mitosis [28]. The results imply that auxin signaling is involved in cell mitosis under submergence. 

When submerged, coleoptiles must mobilize sugar and elongate to enhance the chances of seedling survival. The transporters eventually export sucrose loading and unloading through sucrose transporters or sugars (SWEET proteins) [29]. Many studies have reported that SWEET proteins are involved in plant development and biotic stress. *OsSWEET11* affects pollen development [30], and *OsSWEET14*-knockout mutant reduces the seed size and delays growth [31]. Under abiotic stress, OsSWEET11, OsSWEET13, and OsSWEET14 enhance rice resistance to bacterial blight [31,32]. In this study, four SWEET genes, namely *Os12g0476200* (*OsSWEET13*), *Os11g0508600* (*OsSWEET14*), *Os01g0700100* (*OsSWEET2b*), and *Os01g0606000* (*OsSWEET6a*), were downregulated in rice coleoptiles under SUB + TIBA treatment. We demonstrated that *Os12g0476200* and *Os11g0508600* genes are affected by not only biotic stress, but also auxin signaling under submergence (Table 3 and Figure 4). In addition, auxin signaling might mediate rice coleoptile phenotypes through sucrose transport (SWEET proteins). Furthermore, some transcript levels of mitochondrial electron transport complex II members (*Os10g0516300*, *Os04g0182800*, and *Os03g0835400*) and alternative oxidase members (*Os02g0318100* and *Os04g0600200*) are upregulated in SUB + TIBA treatment (Figure 7A,B). Our results also demonstrated that auxin signaling is involved in the regulation of mitochondria energy production and the AOX signaling pathway in rice coleoptile under submergence.

Taken together, our results presented that auxin signaling was involved in the effect of genes that are related to secondary metabolism, development, carbohydrate metabolism, and mitochondrial electron transport, and also regulated the elongation of rice coleoptiles under submergence. It can provide important information for the breeding program of wet direct-seed system.

## 4. Materials and Methods

### 4.1. Plant Materials and Growth Conditions

We used rice cultivar Taikeng 9 (*Oryza sativa* L. *japonica*; Taiwan) as the plant material in this study. Taikeng 9 has high quality, good taste, and it was one of the famous cultivars in Taiwan. The seeds were removed from the husk to obtain husked rice. The surface of the husked rice was sterilized with 3% sodium hypochlorite for 40 min in a 50-mL centrifuge tube and washed several times with sterile deionized water in laminar flow. After being subjected to cleaning and sterilization, the seeds were imbibed in sterile deionized water and then covered with a black cloth for one day at 28 °C in a 16-h-light/8-h-dark cycle in a growth chamber. For the submergence treatments with (SUB + TIBA) and without TIBA (SUB), the seeds were placed in test tubes (five seeds per tube) after imbibition, and the tubes were filled with a Kimura B solution with or without 10 μM TIBA to reach 10 cm in height. TIBA are used as an auxin polar transport inhibitor [33]. The test tubes were covered with black cloth in a growth chamber for two, three, four, five, and six days.

### 4.2. Measurement Length of Coleoptiles

The coleoptile length was measured while using an ordinary ruler after submergence treatment with or without TIBA for two, three, four, five, and six days, and the lengths of each coleoptile were recorded for the indicated times. The experiments were repeated three times, and at least 30 coleoptiles were measured independently each time. The coleoptiles were collected after five days of treatment, frozen in liquid nitrogen, and then stored at −80 °C for RNA isolation. The submerged coleoptile samples with and without TIBA treatment were analyzed at least six times independently.

### 4.3. Transcriptomic Analysis by RNA Sequencing

The total RNA for each five-day treatment was extracted while using the RNeasy Protect Mini Kit (Qiagen, Hilden, Germany). The RNA concentration was measured using the NanoDrop Lite (Thermo, Waltham, MA, USA), and RNA integrity was assessed while using the RNA Nano 6000 Assay Kit in the Agilent 2100 Bioanalyzer system (Agilent Technologies, Santa Clara, CA, USA). RNA samples with 260/280 ratios greater than 2.0 and RNA integrity numbers greater than 9.0 measured using a bioanalyzer were used. RNA sample quality was required to be greater than 200 ng/µL, and a total amount of 1 µg RNA per sample was used as input material for the RNA sample preparations. Library preparation for RNA-seq was performed while using the NEBNext^®^ UltraTM RNA Library Preparation Kit (San Diego, USA) and followed the manufacturer’s recommendations and index codes were added to attribute sequences to each sample. The final library quality was assessed on the Agilent Bioanalyzer 2100 system while using DNA High Sensitivity Chips. RNA sequencing on an Illumina HiSeqTM 2000 were commercially performed (BGI; https://www.bgi.com/bgi-online) to generate paired-end reads, with each being 100 base pairs in length. Removing the adapters and low quality reads with FASTX-Toolkit was utilized to filter the raw data (http://hannonlab.cshl.edu/fastx_toolkit/index.html). After RNA sequencing was conducted, all of the clean data were calculated while using Q20, Q30, GC-content, and sequence duplication levels and they were compared with the rice reference genome in the Rice Genome Annotation Project Database (MSU7.0, http://rice.plantbiology.msu.edu/)(Table S1).

### 4.4. Functional Analysis of Differentially Expressed Genes

Differentially expressed genes (DEGs) exhibiting 1.5-fold changes and Benjamini–Hochberg adjusted P values (FDR) of < 0.01 were selected. The functions of DEGs were identified while using the Gene Ontology (GO) and Kyoto Encyclopedia of Genes and Genomes (KEGG) databases. GO functional enrichment analysis was performed using Blast2GO (version 2.3.5, BioBam, valencia, Spain) (http://www.blast2go.org/) [34]. The KEGG pathway analyses of the DEGs were performed using the public pathway database (https://www.genome.jp/kegg/pathway.html). Significant genes can be integrated with high-throughput data analysis to produce diverse overviews by using MapMan (http://MapMan.gabipd.org) [35]. The genes were mapped to the OS-Nipponbare-Osa-RAPDB reference genome and used to visualize the involvement of the DEGs in the pathways by using MapMan version 3.6.0RC1.

### 4.5. Extraction of Total RNA and Quantitative Real-Time Polymerase Chain Reaction

The coleoptile samples for each treatment were frozen in liquid nitrogen and ground into powder, and the total RNA was extracted while using TRI Reagent (Invitrogen, MA, USA). For DNase treatment, the total RNA was reacted at 37 °C for 30 min. and at 70 °C for 10 min. to stop the reaction by using the TURBO DNA-free kit (Ambion, Austin, TX, USA). First-strand complementary DNA (cDNA) was synthesized with the Moloney murine leukemia virus (MMLV) first-strand synthesis kit (Gene DireX, Las Vegas, NV, USA). The RNA concentration was spectrophotometrically determined, and the 260/280-nm absorbance ratio exhibited expected values between 1.8 and 2.0. The first-strand cDNA synthesis process was performed, as follows: 2 µg of total RNA was mixed with 1 µL of Oligo dT (1 µg/µL) and 1 µL of 10 mM dNTP, then reacted for 10 min. at 70 °C and for 5 min. at 4 °C. After the reaction, we mixed 4 µL of 5× reaction buffer, 2 µL of 0.1 M dithiothreitol (DTT), 1.75 µL of diethylpyrocarbonate (DEPC) H_2_O, and 0.25 µL of MMLV reverse transcriptase into the solution and reacted it for 1 h at 37 °C and 10 min. at 65 °C. Finally, 80 µL of sterilized deionized water was added to a 0.2-mL microcentrifuge tube for the subsequent quantitative real-time polymerase chain reaction (qRT-PCR) experiment. For the qRT-PCR, the cDNA was amplified while using the CFX ConnectTM real-time system (Bio-Rad, Hercules, CA, USA), and the data were analyzed using Bio-Rad CFX Manager 3.1. The reaction conditions were as follows: 5 min. of predenaturation at 94 °C, 45 cycles of 30 s at 94 °C, 30 s at 55 °C, and 30 s at 72 °C, followed by melting curve generation. Table S2 lists the primers used for qRT-PCR analyses. Primer 3 designed the primers in this study (http://bioinfo.ut.ee/primer3-0.4.0/) [36]. The ubiquitin-conjugating enzyme (*Os02g0634800*) gene was used as an internal control to normalize the cDNA levels. Furthermore, we used the delta-delta CT method to calculate the relative expression level between SUB and SUB + TIBA treatment samples [37]. The experiments were independently repeated at least six times.

## 5. Conclusions

The study results indicate that auxin signaling was involved in the effect of genes that are related to secondary metabolism, development, carbohydrate metabolism, and mitochondrial electron transport in submerged rice coleoptiles growth.

## Figures and Tables

**Figure 6:**
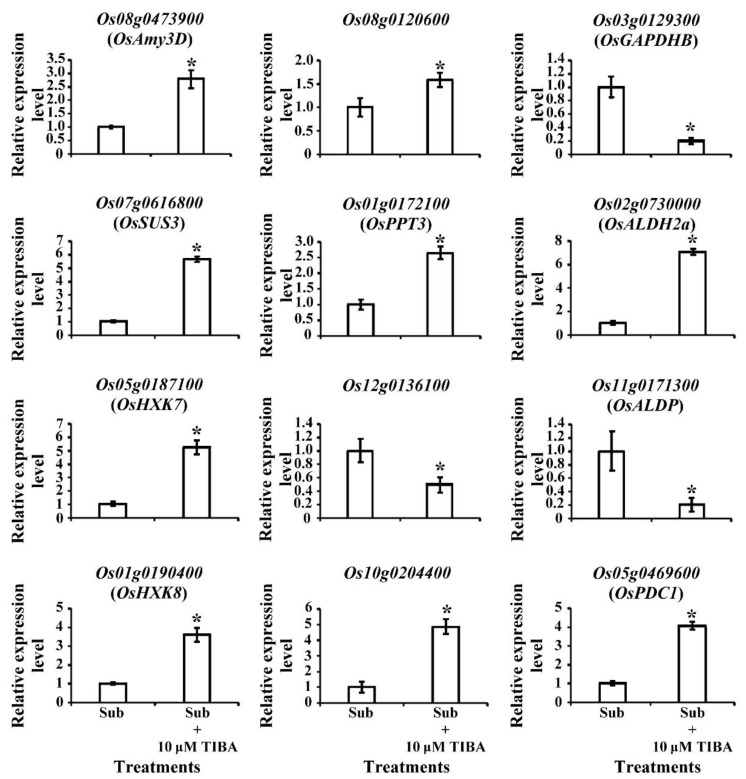
Transcript levels of glycolysis-pathway-associated genes in submerged coleoptiles with 10 μM TIBA. Total RNA was isolated from the coleoptile after 5 days of submergence treatment with or without 10 µM TIBA. The relative expression levels of glycolysis-pathway–-associated genes were determined using quantitative RT-PCR. These genes were calculated and normalized with ubiquitin gene (*Os02g0634800*) as an internal standard. Values are presented as mean ± standard deviation based on 30 coleoptiles of each treatment obtained from six biologically independent experiments. * *p* < 0.05 versus the submergence treatment value (Student’s *t*-test).

**Table 1:** The differentially expressed gene was significantly upregulation between submergence (SUB) and submergence with 10 μM TIBA (SUB + TIBA) in coleoptiles.

GO Function	Gene ID	Name	Description	FPKM		Fold Change	FDR
				SUB	TIBA		
Biotic or abiotic stress	*Os06g0195800*	OsDJC53	DnaJ domain protein	0.01	8.85	885.0	4.9 × 10^−14^
	*Os11g0592200*	OsPR4	Pathogenesis-related protein	0.01	6.27	627.0	8.1 × 10^−8^
Hormone metabolism	*Os11g0453900*	OsRAB16D	Responsive to ABA 16D	0.01	3.15	315.0	3.3 × 10^−5^
	*Os04g0339400*	OsAKR	Aaldo-keto reductase	1.58	387.55	245.3	0.0
Protein degradation	*Os08g0126000*		E3 ubiquitin-protein ligase	0.01	3.51	351.0	1.6 × 10^−15^
	*Os09g0243200*		E3 ubiquitin-protein ligase	0.01	2.7	270.0	8.8 × 10^−4^
Redox	*Os07g0638300*		1-Cys peroxiredoxin	0.01	7.91	791.0	3.1 × 10^−5^
	*Os01g0628000*		Cytochrome P450	0.01	3.85	385.0	8.8 × 10^−6^
	*Os03g0785900*		Glutathione S-transferase	4.97	1269.51	255.4	0.0
	*Os09g0367700*		Glutathione S-transferase	0.63	148.56	235.8	4.5 × 10^−268^
	*Os10g0527400*		Glutathione S-transferase	0.27	54.7	202.6	2.2 × 10^−119^
RNA transcription	*Os06g0719900*		Endo/excinuclease amino terminal domain-containing protein	0.01	6.36	636.0	1.2 × 10^−14^
	*Os05g0223200*		Glycine-rich RNA-binding protein	0.01	4.58	458.0	6.1 × 10^−7^
	*Os05g0404700*		Methyl-CpG-binding domain-containing protein	0.01	2.07	207.0	1.7 × 10^−3^
Secondary metabolism	*Os08g0112300*		Acetyltransferase	0.01	5.07	507.0	7.9 × 10^−16^
	*Os03g0184550*		Dihydroflavonol-4-reductase	0.01	3.98	398.0	2.8 × 10^−9^
Signalling transduction	*Os06g0225000*	OsSAR1d	GTP-binding protein	0.01	5.18	518.0	6.1 × 10^−7^
	*Os04g0655400*		G-type lectin S-receptor-like serine/threonine-protein kinase	0.01	2.65	265.0	1.7 × 10^−5^
Other	*Os09g0324000*		Oleosin	0.01	7.99	799.0	3.6 × 10^−10^
	*Os01g0695800*		MDR-like ABC transporter	0.01	4.90	490.0	2.1 × 10^−8^
	*Os07g0529000*	OsL85	Isocitrate lyase	0.01	4.21	421.0	2.5 × 10^−17^
	*Os05g0366600*		Beta-glucosidase	0.01	3.59	359.0	6.2 × 10^−15^
	*Os02g0586900*		Glycine-rich cell wall structural protein	0.47	112.36	239.1	2.3 × 10^−146^

Gene descriptions were extracted from the NCBI database and MSU Rice Genome Annotation Project Release 7 gene annotation.

**Table 2:** The differentially expressed gene was significantly downregulation between submergence (SUB) and submergence with 10 μM TIBA (SUB + TIBA) in coleoptiles.

GO Function	Gene ID	Name	Description	FPKM		Fold Change	FDR
				SUB	TIBA		
Biotic or abiotic stress	*Os07g0429600*		Tthionin like peptide	2.71	0.01	0.0037	7.4 × 10^−3^
	*Os07g0431160*		Tthionin like peptide	2.68	0.01	0.0037	2.2 × 10^−3^
Carbohydrate metabolism	*Os10g0113900*		NAD(P)H-dependent oxidoreductase	5.09	0.01	0.0020	1.1 × 10^−12^
Cell wall metabolism	*Os10g0545500*		Xyloglucan endotransglucosylase/hydrolase protein	5.04	0.01	0.0020	2.9 × 10^−7^
	*Os01g0946500*		Glucan endo-1,3-beta-glucosidase	2.48	0.01	0.0040	7.3 × 10^−6^
	*Os08g0253800*		Xyloglucan glycosyltransferase	2.07	0.01	0.0048	2.9 × 10^−7^
Development	*Os01g0606000*	OsSWEET6a	Bidirectional sugar transporter	2.49	0.01	0.0040	2.6 × 10^−5^
Hormone metabolism	*Os09g0437100*	OsSAUR37	Small auxin-up RNA	2.86	0.01	0.0035	1.8 × 10^−4^
Lipid metabolism	*Os01g0882100*		Esterase	8.07	0.01	0.0012	7.4 × 10^−3^
	*Os07g0174400*		Lipid transfer protein	7.27	0.01	0.0014	2.6 × 10^−5^
	*Os04g0554500*		Lipid transfer protein	2.98	0.01	0.0034	3.4 × 10^−4^
	*Os11g0299300*		Phospholipase	2.87	0.01	0.0035	3.0 × 10^−9^
Photosynthesis	*Os04g0545600*		Stellacyanin-like protein	8.27	0.01	0.0012	5.7 × 10^−13^
	*Os01g0869800*		Photosystem II 22 kDa protein	4.26	0.01	0.0023	1.1 × 10^−8^
	*Os04g0545400*		Stellacyanin	2.72	0.01	0.0037	4.0 × 10^−3^
	*Os02g0758800*		Blue copper protein	2.00	0.01	0.0050	3.4 × 10^−4^
Protein degradation	*Os06g0278000*		Glucoamylase	8.43	0.01	0.0012	3.9 × 10^−16^
	*Os06g0257600*		Esterase	4.70	0.01	0.0021	5.7 × 10^−13^
	*Os01g0727840*		Subtilisin-like protease	2.20	0.01	0.0045	7.4 × 10^−3^
Protein synthesis and modification	*Os09g0535400*		Proline-rich receptor-like protein kinase	2.18	0.01	0.0046	7.3 × 10^−6^
Redox	*Os12g0263000*		Glutathione synthase	5.69	0.01	0.0018	5.5 × 10^−15^
	*Os03g0339300*		Peroxidase	2.36	0.01	0.0042	7.3 × 10^−6^
	*Os07g0677300*		Peroxidase	2.17	0.01	0.0046	2.6 × 10^−5^
	*Os06g0203200*		Flavin-containing monooxygenase	2.08	0.01	0.0048	4.1 × 10^−8^
RNA transcription	*Os01g0968800*	OsDREB1f	Dehydration-responsive element-binding protein	6.54	0.01	0.0015	2.2 × 10^−10^
	*Os05g0442400*	OsMID1	Myb-like DNA-binding domain containing protein	3.74	0.01	0.0027	1.1 × 10^−6^
	*Os01g0821300*	OsWRKY86	WRKY transcription factor	3.23	0.01	0.0031	5.0 × 10^−5^
	*Os12g0106200*	OsLBD12-1	Lateral organ boundaries domain (LBD) family transcription factor	2.90	0.01	0.0034	6.3 × 10^−4^
	*Os09g0414500*		Mini zinc finger protein	2.59	0.01	0.0039	2.2 × 10^−3^
	*Os02g0781300*	OsERF11	Ethylene-responsive transcription factor	2.53	0.01	0.0040	6.3 × 10^−4^
	*Os04g0590800*	OsbHLH94	Basic helix-loop-helix protein	2.40	0.01	0.0042	2.2 × 10^−3^
	*Os09g0522200*		Dehydration-responsive element-binding protein	2.30	0.01	0.0043	3.4 × 10^−4^
	*Os09g0538000*		Ribonuclease	2.04	0.01	0.0049	9.5 × 10^−5^
Secondary metabolism	*Os04g0609300*		Shikimate O-hydroxycinnamoyltransferase	2.01	0.01	0.0050	2.0 × 10^−6^
Signaling transduction	*Os06g0339500*		LRR receptor-like serine/threonine-protein kinase	2.55	0.01	0.0039	7.4 × 10^−3^
	*Os11g0105000*	OsCML25	Calcium-binding protein	2.24	0.01	0.0045	7.4 × 10^−3^
	*Os12g0104900*	OsCML26	Calcium-binding protein	2.24	0.01	0.0045	7.4 × 10^−3^
Transport	*Os05g0338933*	OsNRT1	Proton-dependent oligopeptide transporter	4.07	0.01	0.0025	4.0 × 10^−3^
	*Os02g0658100*	OsTIP2	Aquaporin	3.60	0.01	0.0028	7.3 × 10^−6^

Gene descriptions were extracted from the NCBI database and MSU Rice Genome Annotation Project Release 7 gene annotation.

**Table 3:** The differentially expressed genes of development responsive were compared with submergence (SUB) and submergence with 10 μM TIBA (SUB + TIBA) in rice coleoptiles.

Log2 (FC)	Gene ID	Name	Description	FPKM		Fold Change	FDR	
				SUB	TIBA			
	*Os06g0273200*		Trichome birefringence-like protein	0.01	1.54	154.0	4.6 × 10^−4^	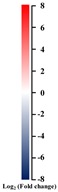
	*Os01g0306400*	OsDOG1L-3	Delay of germination 1-like protein	0.01	1.37	137.0	4.6 × 10^−4^	
	*Os02g0616600*		Meiosis arrested at leptotene like protein	0.85	87.01	102.4	0.0	
	*Os01g0762500*	OsGLUA-1	Glutelin type-A-1	0.12	2.25	18.7	3.7 × 10^−6^	
	*Os01g0606000*	OsSWEET6a	Bidirectional sugar transporter	2.49	0.01	0.004	2.6 × 10^−5^	
	*Os09g0307300*		Microtubule-associated protein	1.83	0.01	0.005	1.8 × 10^−4^	
	*Os03g0578500*		Myosin	1.95	0.01	0.005	1.2 × 10^−3^	
	*Os08g0477500*	OsPLP2	Patatin-like protein	1.54	0.01	0.006	7.3 × 10^−6^	
	*Os06g0234600*	OsTBL52	Trichome birefringence-like protein	1.53	0.01	0.007	9.5 × 10^−5^	
	*Os01g0803300*		Walls are thin1 (WAT1)-related protein	1.26	0.01	0.008	2.2 × 10^−3^	
	*Os09g0375300*		Trichome birefringence-like protein	1.12	0.01	0.009	4.0 × 10^−3^	
	*Os03g0291200*		Trichome birefringence-like protein	1.04	0.01	0.01	2.2 × 10^−3^	
	*Os01g0281200*	OsCYCB1-3	G2/mitotic-specific cyclin-B1-3	0.77	0.01	0.013	7.4 × 10^−3^	
	*Os08g0443800*	OsTET12	Tetraspanin	4.96	0.15	0.03	7.1 × 10^−9^	
	*Os07g0671000*		Flowering-promoting factor 1-like protein	24.62	0.83	0.034	6.1 × 10^−15^	
	*Os01g0563000*	OsFKBP73	Peptidyl-prolyl cis-trans isomerase	77.11	2.59	0.034	3.0 × 10^−17^	
	*Os01g0968400*		Root cap family protein	5.33	0.26	0.049	8.8 × 10^−10^	
	*Os05g0557400*	OsNSL1	Membrane attack complex/perforin (MACPF) domain containing protein	2.08	0.13	0.063	2.1 × 10^−7^	
	*Os07g0691700*	OsWAVE5	Suppressor of cAMP receptors (SCAR)-like domain containing protein	0.76	0.05	0.066	7.5 × 10^−4^	
	*Os11g0508600*	OsSWEET14	Bidirectional sugar transporter	7.58	0.51	0.067	4.0 × 10^−16^	
	*Os05g0563000*		NAC transcription factor	1.45	0.1	0.069	1.3 × 10^−3^	
	*Os12g0476200*	OsSWEET13	Bidirectional sugar transporter	9.44	0.65	0.069	4.9 × 10^−22^	
	*Os01g0172800*		Embryo-specific 3 family protein	35.49	2.47	0.07	7.6 × 10^−40^	
	*Os04g0422300*		Walls are thin1 (WAT1)-related protein	1.39	0.1	0.072	2.3 × 10^−3^	
	*Os01g0700100*	OsSWEET2b	Bidirectional sugar transporter	1.55	0.13	0.084	7.1 × 10^−3^	
	*Os03g0229600*		MFP1 attachment factor 1 (MAF1)-like protein	1.47	0.13	0.088	4.0 × 10^−5^	
	*Os06g0210000*		Walls are thin1 (WAT1)-related protein	2.73	0.24	0.088	7.1 × 10^−5^	
	*Os06g0236600*	OsCYCD1-1	G1/S-specific cyclin-D1-1	3.16	0.28	0.089	7.1 × 10^−5^	
	*Os07g0601600*		3-oxo-delta(4,5)-steroid 5-beta-reductase	2.71	0.24	0.089	7.1 × 10^−5^	

The log2 fold change color scale ranges from −8 to 8. Gene descriptions were extracted from the NCBI database and MSU Rice Genome Annotation Project Release 7 gene annotation.

## Supplementary Materials

Supplementary materials can be found at https://www.mdpi.com/1422-0067/21/4/1292/s1.

## Abbreviations

SUB	Submergence
TIBA	2,3,5-triiodobenzoic acid
DEGs	Differentially expressed genes
qRT-PCR	Quantitative real time polymerase chain reaction
