# Supplementary material for: Comprehensive Transcriptomic Analysis of Auxin Responses in Submerged Rice Coleoptile Growth
Source: Int J Mol Sci. 2020 Feb 14;21(4):1292. doi: 10.3390/ijms21041292 (PMC7072898; doi:10.3390/ijms21041292)
Supplement: Supplementary file 1 [file ijms-21-01292-s001.zip › ijms-702709-supplementary 3/SUP/TableS1.docx]

Supplement table1 Short reads alignment to MSU Rice Genome Annotation Project Release 7 assembly and the quality.

| RNA Seq library | Total Reads | Total  Mapped Reads | (%) | Unique Match | (%) | Multi-position Match | (%) | Total Unmapped Reads | (%) | Q20  (%) | Q30  (%) | GC content  (%) |
| --- | --- | --- | --- | --- | --- | --- | --- | --- | --- | --- | --- | --- |
| SUB | 13,009,199 | 11,338,976 | 87.16 | 10,526,796 | 80.92 | 812,180 | 6.24 | 1,670,223 | 12.84 | 99.05 | 97.10 | 54.38 |
| 10μM TIBA | 13,006,825 | 11,213,701 | 86.21 | 10,366,278 | 79.7 | 847,423 | 6.52 | 1,793,124 | 13.79 | 99.06 | 97.14 | 53.95 |
