# Supplementary material for: Comprehensive Transcriptomic Analysis of Auxin Responses in Submerged Rice Coleoptile Growth
Source: Int J Mol Sci. 2020 Feb 14;21(4):1292. doi: 10.3390/ijms21041292 (PMC7072898; doi:10.3390/ijms21041292)
Supplement: Supplementary file 1 [file ijms-21-01292-s001.zip › ijms-702709-supplementary 3/SUP/TableS2.docx]

Supplement table 2 The primer sequence used for quantitative RT-PCR in this study.

| **GeneID** | **Name** | **Forward sequence (5'→3')** | **Reverse sequence (5'→3')** |
| --- | --- | --- | --- |
| Os02g0634800 |  | AAGGC ATGGC TGATT CATGT | CAGAT GTTCA CATGG TGACA GTA |
| Os06g0236600 | OsCYCD1-1 | GAGCT CGAGT TCCTG AACCA | TGCAG GCGAC TCATT TACTG |
| Os01g0281200 | OsCYCB1-3 | CATCG CTTTG ATGTT ACCCC | TCCAA TCAAA TCAGG AACCC |
| Os12g0476200 | OsSWEET13 | CTACG GCCTT CTCAT CAAGG | GAATG TGAAG CCCAG GATGT |
| Os01g0700100 | OsSWEET2a | TGATT CCCTC CTTTT CCTTG | TGAGG CTCAA TGTGC ATAGC |
| Os11g0508600 | OsSWEET14 | TGTGG ATCTA CTACG CGCTG | ACGGC GATGT AGATG GTCTC |
| Os01g0606000 | OsSWEET6b | CCCAA CAGTA TCCTC GTCGT | CGTTT CTTGT TGGGG GAGTA |
| Os08g0473900 | OsAmy3D | GACTACAGCGTCTGGGAGAAG | TGTAGAAACTGGTTTGAGCATGACT |
| Os08g0120600 |  | GAGAA TGTCG AGGAG AACCG | CTCGA ACAGG ATGAC GCC |
| Os03g0129300 |  | GAAGG GTGCT GACAT CCCTA | TGATA ATGTT GGCCA CTTCG |
| Os07g0616800 | OsSUS3 | CCTGG CCTTT ATCGT GTTGT | CATGT CAGCA CCAGG AGAGA |
| Os01g0172100 | OsPPT3 | ATCGT GGCGT CTGTT CTCTT | AACTC CTCCC AATGC AACAC |
| Os02g0730000 | OsALDH2a | CTACA TCCAG CCAAC CGTCT | ACTTG AGGAT GGACT GCACC |
| Os05g0187100 | OsHXK7 | GGGTT CACCT TCTCT TTCCC | TAGAA AACGC CTTGG TCCAC |
| Os12g0136100 |  | AAAGCTTCCGCTCCTACCTC | CAGTAGCACCGACTTGAGCA |
| Os11g0171300 | OsALDP | CGACA TCCTC ACTGA GCAGA | GAGAC CTTGG CACCA TGACT |
| Os01g0190400 | OsHXK8 | CAAAG GCGTT TTCCA TTGAT | CCTTG TTTCA CCATA GCCGT |
| Os10g0204400 |  | TGGGA ACACA ATATG CTGGA | CGAGA GGATC TGCTT TTTGG |
| Os05g0469600 | OsPDC1 | TGTTTCTGTATCCATTCAAAATTTTCTT | CATTTGAATTACATACATTATCATAGGAT |
| Os08g0141400 |  | AACAA GAAGA TCCAT TGCCG | GTCAA CCAAG AATTC GCCAT |
| Os04g0182800 |  | GTGGA GAGAG AGGTC GATGG | TGCAT ACCTT GGTTG GTTCA |
| Os02g0318100 |  | AGAGG AATCG GAGAA GGAGG | AAGCA GGACC ACTTC CACAC |
| Os07g0564500 |  | AAGCT GCTCA CCAAC CTCAT | AGGCG CTTCT TCTCT TCCTC |
| Os03g0835400 | OsETFA | GCGCT TTGTA CTGTG CGATA | CATAG CCTCA GTTGG GGAAA |
| Os04g0600200 | OsAOX1a | AAGGA GGTGG TGGTC AACAG | CAAGA CCACT TCCAC TCCGT |
| Os07g0621600 |  | AGCAG CTCAG GACAC CACTT | CTCCC CTCTT CTTCT GGCTT |
